# Management factors affecting milk yield, composition, and quality on smallholder dairy farms

**DOI:** 10.1007/s11250-025-04294-x

**Published:** 2025-01-29

**Authors:** Marie Anne Mukasafari, Jean Pierre Mpatswenumugabo, Jean Baptiste Ndahetuye, Ewa Wredle, Renée Båge

**Affiliations:** 1 Department of Applied Animal Science and Welfare, Swedish University of Agricultural Sciences, Box 7024, 750 07 Uppsala, Sweden; 2 Department of Clinical Sciences, Swedish University of Agricultural Sciences, Box 7024, 750 07 Uppsala, Sweden; 3 Department of Veterinary Medicine, University of Rwanda, Box 57, Nyagatare, Rwanda

**Keywords:** Milk quality, Antibiotic residues, Feeding practices, Dairy cattle, Milking procedures

## Abstract

A cross-sectional study on 156 smallholder dairy farms in Rwanda was carried out to assess the association between farm management practices and milk yield and quality. A pre-tested questionnaire was used to collect data on cow characteristics and farm management practices. Milk yield was recorded at household level, milk composition was monitored using a Lactoscan device (Milk Analyzer). Somatic cell count (SCC) was determined using a DeLaval cell counter (DCC). A Delvotest SP-NT kit was used to determine antibiotic residues in raw milk. Most dairy cows were kept in zero-grazing system (84.6%) and most farmers had less experience of dairy production (78.2%). Mean daily milk yield was 3.9 L/cow and was associated with type of breed, milking frequency, stage of lactation, and parity. Mean milk content of protein, fat, lactose and solid non-fat, and density were normal and showed no association with different management practices. Based on SCC analyses, 65.8% of the milk samples with less than 300,000 cells/mL were graded as acceptable for delivery to a milk collection centre (MCC) and 12.9% of the samples tested positive for antibiotic residues. These findings suggest low milk yields on smallholder farms in Rwanda that are attributable to type of breed and prevalent high level mastitis, among other factors. The results also indicate possible non-compliance with withdrawal periods, resulting in antibiotic residues in milk, which has public health implications for consumers. Routine testing at MCC for both SCC and antibiotic residues is important for quality control.

## Introduction

Milk is a major animal-source food (ASF) that can play a crucial role in alleviating poverty and improving human nutrition, health and well-being (FAO et al. 2022). Milk is a highly nutritious and valuable source of fats, amino acids, minerals and vitamins that form part of the recommended daily intake for humans. Essential nutrients found in milk are known to be important for the growth and cognitive development and health maintenance of young children (McMahon 2016; Beal et al. 2023). ASF provides more essential nutrients like calcium, proteins and vitamin B12 than most plant-based sources, which can be particularly beneficial for young children and pregnant mothers (Beal et al. 2023). Milk yield, composition, and quality aspects are also important for the milk processing industry. However, with the increasing trend of the human population, particularly in sub-Saharan Africa, there has been a rise in the demand for ASF as reported by Bateki et al. (2020). The insufficient consumption of ASF, such as milk, has been linked to high incidences of stunting in young children (McMahon 2016). In order to meet the demand for milk and prevent stunting, both milk quality and production must improve. In tropical regions, however, the productivity of dairy cattle remains low. This is primarily due to the scarcity of feed, both in terms of quantity and quality, particularly during the dry season. In sub-Saharan Africa, feeding strategies heavily rely on natural pastures during the wet season, followed by the utilization of crop residues and green forages without supplements decrease dairy performance (Duguma and Janssens 2016; Ramírez-Rivera et al. 2019). Water scarcity and drought in tropical harsh conditions adversely affects milk yield, composition and quality by reducing intake and interfering with their metabolism due to heat stress (Hernández-Castellano et al. 2019).

Milk composition and quality are also influenced by several factors, such as animal breed, stage of lactation, parity (Gustavsson et al. 2014), and management practices such as feeds and feeding system (Kashongwe et al. 2017; Mayberry et al. 2017). In tropical countries, local breeds produce less milk compared to improved crossbreeds of pure and local cattle (Ramírez-Rivera et al. 2019; Bateki et al. 2020). This observation is consistent with findings from Rwanda, where the local Ankole breed produces less milk than improved crossbreeds (Manzi et al. 2020). However, the performance of improved breeds in tropical regions is often affected by unpredictable weather conditions, such as drought and high temperatures, which can reduce feed intake and overall productivity. Milk production is influenced by the animal health status. Intramammary infections, manifested as high SCC levels, reduce milk yield as a result of subclinical or clinical mastitis (Hagnestam-Nielsen et al. 2009). Milk quality is also negatively affected by mastitis when its causing pathogens attack and reduce numbers in milk producing cells in the mammary gland, e.g. according to Ma et al. (2000), intramammary infections affect milk quality by increasing proteolysis and lipolysis of milk components, thus reducing its shelf-life. Farm hygiene also has an impact on milk quality, with poor cleaning of cow shelters increasing infection pressure on the farm, therefore, disease incidence (Garcia et al. 2023) and affecting udder health (Ndahetuye et al. 2020b).

Furthermore, potential pathogens associated with intramammary infections, such as *Staphylococcus* spp., *Escherichia coli*, *Campylobacter jejuni* etc., can cause food poisoning (Petzer et al. 2017) and a number of health hazards such as chronic reactive arthritis (Mor-Mur and Yuste 2010), meningitis and abortions (D’Angelo et al. 2022). Antibiotics are often used to lower SCC in herds with udder health problems, posing a risk of the presence of antibiotic residues in ASF if withdrawal times are not respected (Alves et al. 2020). A recent study confirmed problems with antibiotic residues in ASF as a result of misuse of antimicrobials in animals (Chowdhury et al. 2021). Health risks associated with antibiotic residues in milk include development of antimicrobial resistance (AMR), hypersensitivity reactions, and cancer (Rahman et al. 2021). Developing countries are at greater risk than developed countries due to poor detection facilities, lack of proper monitoring systems and permissible thresholds for antimicrobial residues in foods (Pokharel et al. 2020). Poor antimicrobial stewardship programmes and lack of knowledge about appropriate antimicrobial use among smallholder farmers in developing countries are the main causes of high levels of antibiotic residues in ASF (Chattopadhyay 2014).

Enhancing the quality of dairy production in smallholder farms within tropical regions presents one of the most fundamental challenges in livestock management. The gaps in dairy nutrition, breeding, health, and farm management practices vary among smallholder farmers across different production systems and countries (Hernández-Castellano et al. 2019). Addressing these gaps requires a deeper understanding of the management practices associated with milk production and quality. However, information on the effects of management practices on milk yield, composition, and quality among smallholder dairy farms is the case in many African countries, is still lacking. The aim of this study was therefore to identify dairy farm management practices affecting milk production, composition, and quality on smallholder dairy farms in Rwanda.

## Material and methods

### Study design and study area

**Fig. 1:**
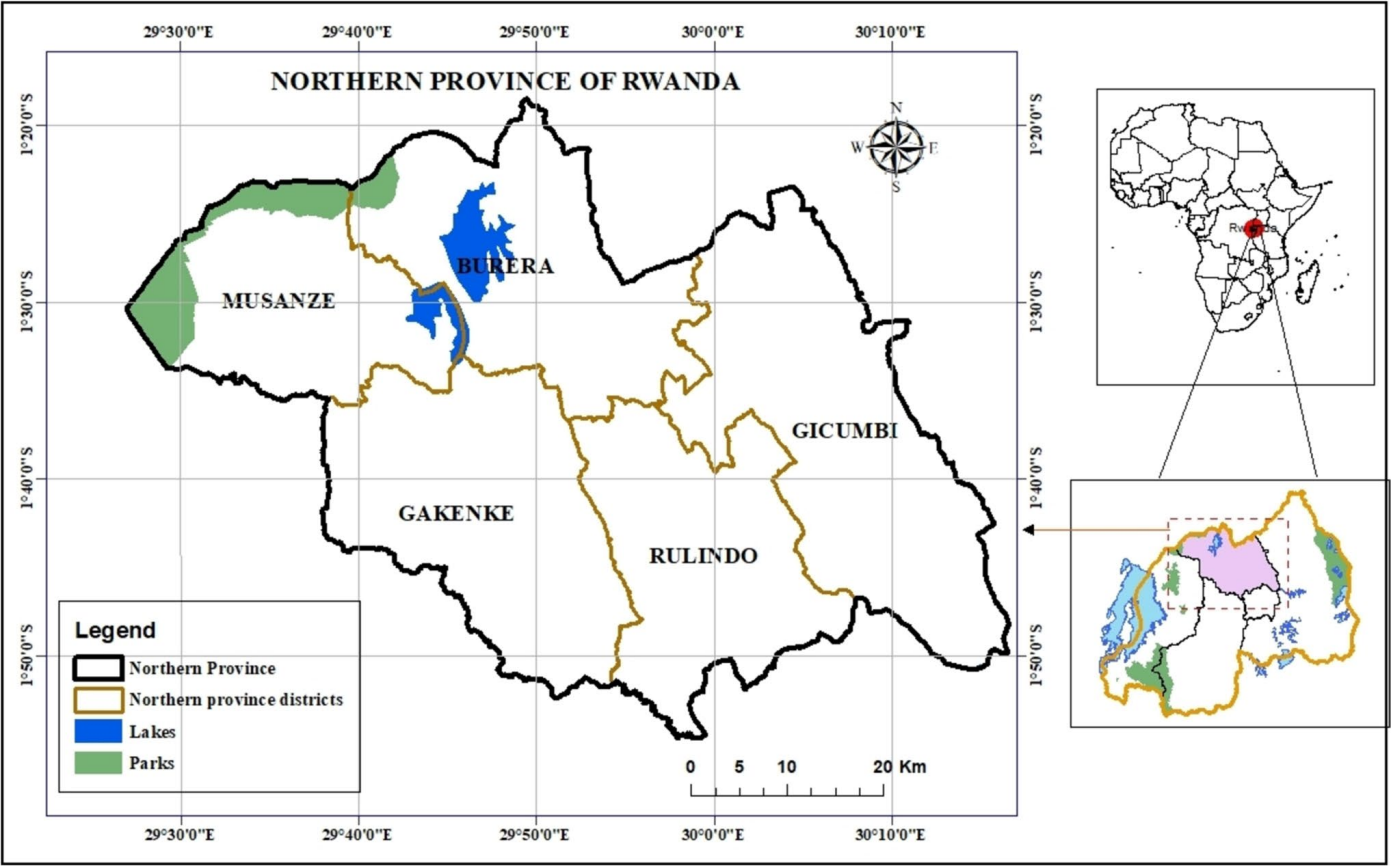
Administrative map showing the Northern Province, Rwanda

The study formed part of a large interdisciplinary research project to combat under-nutrition in children under 3 years and their mothers in the highlands of Northern Province, Rwanda (Fig. 1). Rwanda is located in Subsaharan Africa at 121 km south of the equator, within the Tropic of Capricorn, 1416 km to the west of the Indian Ocean, and 1250 km to the east of the Atlantic Ocean (Rwanda Directorate General of Immigration and Emigration 2016). The Northern province lies at around 2,500 m above sea level and enjoys the coolest weather and most abundant precipitation of all provinces in Rwanda (MINALOC 2021). However, according to the Rwanda Demographic Survey, the province has the highest rate (41%) of stunting in children under 5 years (NISR 2021). A survey, field observations, and laboratory analyses were used in this study to collect relevant information about management factors associated with milk composition and quality on smallholder farms.

### Household selection and smallholder dairy farm identification

Household recruitment was done based on a sampling frame provided by the Ministry of Health. This comprised a list of all households with a child below the age of 3 and a mother aged 18 years and above from each village. One day before the field visit, village leaders and community health workers were contacted by phone to inform them about the study. On the day of data collection, village leaders and community health workers assisted in randomly selecting households to ensure representativeness of the sample size. Briefly, the number of households chosen from each village was determined proportionally to its size. In total, 601 households fulfilled the selection criteria and 156 (26%) of these had lactating cows and were included in the current analysis.

### Farm characteristics and managment factors

A structured questionnaire was designed, pre-tested and adapted. It was installed on an android platform (tablets) to facilitate data collection, storage and quality checks. Prior to data collection, the survey questionnaire was pre-tested on 15 households, of which 6 had lactating cows, and minor adjustments were made. The questionnaire contained an initial section asking for basic household data and another section focusing on feeding practices, milk yield, animal health, milk handling practices, record keeping etc. In addition, field observations were conducted by two of the authors (MAM and JPM) on animal hygiene, milking procedures, milking place and cow body condition score (BCS), as indicated in Table 1.

**Table 1:** Definition of variables used in multivariate analysis and other farm management practices adopted by smallholder dairy farmers in the study area

Variable name	Definition
Response variable	
Milk composition	Continuous variable: fat (%), lactose (%), protein (%),solids-non-fat (SNF) (%) and density (kg/m^3^)
Milk yield	Continuous variable, number of liters of milk produced per cow/day
Somatic Cell Count (SCC)	Continuous variable, number of inflammatory cells per milliliter of milk (cell/mL)
Antibiotic residues	Binary variable, 0 = absence of antibiotic residues in milk, 1 = presence of antibiotic residues in milk
Independent variables	
Household land size	The total land owned by farmers is measured in hectares (Ha). Individuals who did not possess land of their own were categorized as landless, indicating that they were renting their agricultural plots
Farm herd size	The number of total cows per farmer, including calves and bulls
Keeping farm records	The practice by which farmers keep records of farm activities including animal treatment, milk yield, fertility among others
Feeding system	Categorical variable, 1 = Zero grazing: animals are kept in a shed and feeding by cutting and carrying forage and crop residues to the cows, 2 = open grazing: animals freely graze on individual or communal grazing lands, 3 = Semi-grazing which is a hybrid between open-grazing and zero-grazing where animals are kept in a shed but also allowed to graze on nearby land part of the time
Source of forage	The source of forages refers to where farmers get grasses to feed their cows. Some farmers were (1) purchasing forages from neighbours, (2) collecting forages along roadsides or other areas accessible to them, or (3) purchasing and collecting at the same time
Screening for mastitis	Binary variable, 1 = Yes: those who screen for subclinical mastitis, 0 = No: those who do not screen
Source of veterinary services	Categorical variable, 1 = Private Veterinarians: these veterinarians operate independently and have mobile clinics. They offer veterinary services on-call to farmers, 2 = Sector Animal Resources Officer (SARO): these are government officials who occasionally provide veterinary services to farmers, 3 = Farmer Treatment: some farmers opt to treat their own animals, either due to financial constraints or limited access to veterinary services in their area
Previous experience in farming practice	Binary variable, 1 = Yes: those who have been practicing dairy farming at least for three (3) years, 0 = No: those who started dairy farming within the last 3 years
Breed	Categorical variable, 1 = Ankole: a local zebu breed, characterized by low milk yield, 2 = crossbred: a result of crossing the local breed with pure breed (Ankole x Holstein–Friesian/Jersey), 3 = purebred: Holstein–Friesian or Jersey cows
Parity	Categorical variable, 1 = Primiparous: cows that have calved between 1 – 2 times, 2 = Multiparous: cows that have calved > 2 times
Body condition scores (BCS)	Categorical variable, 1 = Backbone prominent (poor); 2 = backbone visible (moderate); 3 = hipbone visible faintly (good); 4 = hipbone not visible (fat); 5 = hipbone showing fat deposit (very fat)
Stage of lactation	Categorical variable, 1 = Early stage: cows that calved within the last 2 months, 2 = middle stage: cows that calved within the last 2 to 6 months, 3 = cows that calved within > 6 months
Milking procedures	Milking procedures refer to the methods farmers employ when milking their cows. These included: 1 = Washing hands before milking; 2 = washing the udder with clean water; 3 = teat dipping (before/after milking); 4 = None (when no pre-milking procedure is done)

### Milk sampling and analyses

It was initially hypothesized that some farmers would have more than one lactating cow at the time of data collection. Based on this assumption, collecting bulked milk samples was anticipated. However, during data collection, we discovered that all participating households had only one lactating cow at the time of the study. Therefore, milk was directly collected from the on-farm milk storage container in 15-mL sterile screw-top tubes that were placed in a cool box (at 4 °C), stored at −20 °C in a freezer at the district hospital until the end of the week and later transported to the University of Rwanda, Busogo campus, for storage at −80 °C for three months until laboratory analyses.

Milk samples were analysed for SCC immediately upon sampling on the farm, using a portable DeLaval cell counter (DCC) (DeLaval International AB, Tumba, Sweden). The results were obtained in cells/µL and converted into cells/mL (Kandeel et al. 2018).

For laboratory analyses, frozen milk samples were thawed and kept at room temperature for around 20–30 min before analysis. For milk composition analysis, the tubes were shaken gently to avoid foam formation and any cream adhering to the tube was removed. Milk composition was analysed using a Lactoscan Milk Analyzer (Milkdata.in, Bangalore, India). Prior to analysis, the machine was calibrated using ultra-high-temperature (UHT) milks with known high and low fat content. The analysis included fat (%), protein (%), solids-non-fat (SNF) (%), density (kg/m^3^) and lactose (%), and was performed according to the manufacturer’s instructions.

Before antibiotic residue testing, the milk samples were gently homogenised in an electrical vortex machine. Testing was performed with a Delvotest SP-NT kit (DSM, Delft, the Netherlands) according to the manufacturer’s instructions. Positive and negative controls were prepared as described previously (Ondieki et al. 2017). For each milk sample (or control), a total of 100 µL milk was placed on the surface of the agar. The plates were then sealed, placed in a water bath and incubated at 64 ± 2 °C for 3 h. Test results were interpreted visually as ‘negative’ (yellow agar) or ‘positive’ (purple agar).

### Statistical analysis

Survey responses were extracted from the survey software and exported to an Excel worksheet (Excel, 2016, Microsoft Corp.). Each response was then assigned a categorical code for subsequent descriptive statistical analysis (Table 1). Results for SCC, milk composition, and antibiotic residue tests were recorded in the same worksheet to facilitate inferential statistical analysis. Cleaned data were exported to R version 4.3.3 (R Core Team, Vienna, Austria) for descriptive and multivariate analyses. Descriptive analyses focused on the percentages of each variable. Multivariate analysis of variance (MANOVA) tests were conducted to identify any potential associations between outcome variables and independent variables, with statistical significance set at *p* < 0.05. Preliminary statistical analysis involved formulating relevant hypotheses related to dependent variables, including milk yield, milk composition, antibiotic residues and SCC and checking for important assumptions. The following formula, modified from previous reference (James et al. 2023), was used to construct the MANOVA models in R.

$${Y}_{i}={x}_{i}{,}_{1}{\beta }_{1}+{x}_{i},{ }_{2}{\beta }_{2}+\cdots +{x}_{i,d}{\beta }_{d}+{e}_{i}={\beta }^{T}{x}_{i}+{\varepsilon }_{i}$$

where i = 1, …, *n* has m ≥ 2 response variables Y_1_, …, Y_m_ and *d* predictor (independent) variables X_1_, X_2_, …, X_d_. Furthermore, *β*_*1*_ through *β*_*d*_ are the unknown coefficients for the predictor variables. Response variables in each model were combined by *cbind()* function to create *Y*_*i*_ matrices.

For the Model 1, Y_1_ is milk yield and Y_2_ is milk composition (fat, protein, lactose, SNF and density) whereas X_1_ through X_8_ are stage of lactation, milking frequency, breed, parity, BCS, feeds, SCC and feeding systems, respectively. In this model, SCC values were categorized into categorical variables based on the threshold stated in East African Community Standards, where SCC < 300,000 cells/mL is deemed acceptable and higher values are deemed unacceptable (COMESA 2006). For the Model 2, Y_1_ is antibiotic residues and Y_2_ is SCC whereas X_1_ through X_7_ are stage of lactation, milking frequency, breed, parity, screening for subclinical mastitis, source of veterinary services and feeding system, respectively. To evaluate the possibility of multicollinearity among independent variables, a Pearson correlation analysis was conducted for all independent variables. There was a weak positive correlation between milking frequency and stage of lactation (= 0.022). However, since stage of lactation did not demonstrate statistical significance in the full model (*p* = 0.855), it was excluded from the subsequent analysis.

Initial analysis comprised a full model to assess any association between response variables and independent variables. In the subsequent phase, a multivariate pairwise comparison analysis was conducted considering independent variables that had a significant association with milk yield. Pillai’s Trace test was selected for its robustness (Warne 2014) in determining the influence of each independent variable on the outcome variable. Both Pillai’s Trace and partial eta squared (η^2^_*p*_) tests were used to evaluate the magnitude of within and between subjects for each independent variable and the proportion of the total variance in the dependent variables (Lakens 2013; Richardson 2011).

## Results

### Farm characteristics and management practices

Descriptive characteristics on the participating smallholder dairy farmers and management practices are presented in Table 2. Over two-thirds (67.1%) of the smallholder farmers were landless, with no possibility to grow forages, and hence most (92.3%) depended on forages collected in different places, such as communal land, open space, playgrounds etc. Most farmers kept their cattle within the homestead and average cattle ownership was around two per farm. A majority of the farms (87.3%) had crossbreed cows and exotic breeds were only kept by 4.6% of the farms. Less than 60% of the farmers kept records, which mainly consisted of fertility records, while 41% did not keep any records. Although nearly 80% had no previous experience in animal rearing, most had adopted some good milking practices at farm level, such as screening for mastitis (62.2%) using California Mastitis Test (CMT) and washing hands and cow udder before milking (92.9%) (Table 2).

**Table 2:** Farm management practices and their respective percentages in the study area (*n* = 156)

Variable	Percentage of farmers
HH land size	
Landless	67.1
1–5 Hectares	29.5
> 5 Hectares	3.4
HH herd size	
1–2 cattle	79.9
3–5 cattle	20.1
Keeping farm records	
Yes	59.2
No	40.8
Feeding system	
Zero grazing	84.2
Semi-zero grazing	15.8
Source of forages	
Purchase	3.8
Collect	40.4
Both	55.8
Screen for mastitis	
Yes	63.8
No	36.2
Source of veterinary services	
Private veterinarians	63.2
Sector Animal Resources Officer (SARO)	32.2
Treatment by the farmer	4.5
Previous experience in rearing dairy cattle	
Yes	21.1
No	78.9
Cow breed	
Ankole (local breed)	7.9
Cross-breed	87.5
Friesian	1.3
Jersey	3.3
Parity	
Primiparous	86.2
Multiparous	13.8
Body condition score (BCS)	
Good	26.3
Moderate	62.5
Poor	11.2
Stage of lactation	
Early (1–2 months)	26.3
Middle (2–6 months)	52.6
Advance (7 months and over)	21.1
Milking procedures	
Washing hands before milking	5.8%
Washing the udder with clean water	88.5%
Teat dipping	3.8%
None	1.9%

### Somatic cell count and antibiotic residues

The mean SCC count was 470,907 cells/mL milk (Table 3) while 34.2% of milk samples had SCC above 300,000 cells/mL, and 19 samples (12.9%) tested positive for antibiotic residues. There were no associations between management practices and SCC or antibiotic residues. However, the distribution of SCC levels according to cow age showed that 77.7% of cows with SCC < 300,000 cells/mL were younger than 6 years, compared to 22.3% that were older than 6 years. A similar pattern was observed in cows with SCC > 300,000 cells/mL.

**Table 3:** Summary statistics for milk yield, composition, and somatic cell counts

Descriptive statistics				
Variables	Minimum	Mean	Maximum	SEM
Milk yield (L/cow/day)	0.5	4.0	12.0	0.19
Fat (%)	1.1	3.1	8.1	0.10
Protein (%)	2.0	3.3	4.8	0.03
Lactose (%)	3.2	5.0	6.7	0.33
Solid not fat (%)	6.7	9.2	12.3	0.06
Density (Kg/m3)	1,022	1,033	1,045	0.20
Somatic cell count (cell/mL)	101,000	470,908	2,516,000	44,888

*SEM* standard error of the mean

### Management factors associated with milk yield and milk compostion

**Fig. 2:**
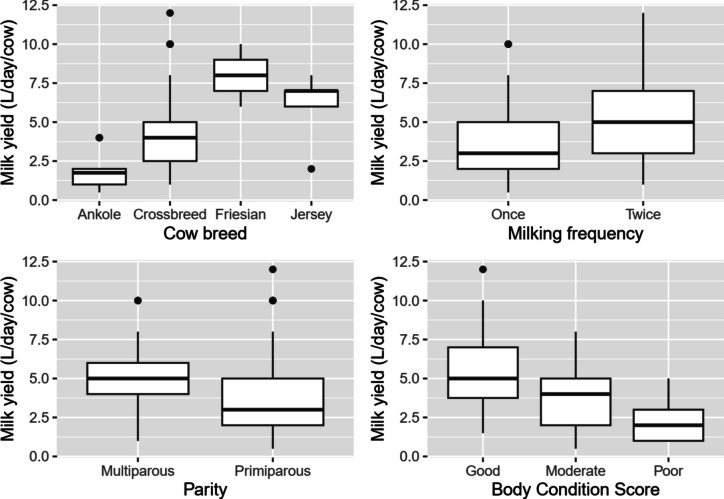
Average daily milk yield associated with breed, milking frequency, parity, and BCS (*p* < 0.05)

Results from Model 1, which included milk yield and milk composition, demonstrated that milking frequency, breed, parity, and body condition score (BCS) were statistically significant (*p* < 0.05). By splitting the R output, it was found that all these variables were only associated with milk yield at various levels (see Table 4). BCS was highly associated with milk yield (*p* = 0.001), followed by breed (*p* = 0.002), milking frequency (*p* = 0.005) and parity showed a weaker relationship with milk yield (*p* = 0.02). Milking twice resulted in a higher milk yield (5.05L ± 2.43, *p* < 0.001) compared to milking once (3.54L ± 1.98), whereas the Ankole breed was associated with a lower overall mean milk yield (1.88L ± 1.11, *p* < 0.001) compared to other breeds. Similarly, good BCS was associated with higher milk yield (5.36L ± 2.60, *p* < 0.001) compared to moderate and poor BCS (*p* > 0.05), while multiparous cows produce more milk (4.98L ± 2.56, *p* = 0.002) than primiparous cows (Fig. 2).

**Table 4:** Assessment of association between farm management practices and milk yield and composition

Independent variable	Pillai value	F-value	df (num, den)	*p*-value
Stage of lactation	0.99	0.7	(18, 390)	0.771
Milking frequency	0.13	32.7	(6, 128)	0.005**
Breeds	0.29	22.7	(18, 390)	0.002**
Parity	0.11	225.4	(6, 128)	0.023*
Body condition score	0.22	27.1	(12, 258)	0.002**
Feed used	0.17	1.0	(24, 524)	0.506
SCC groups	0.18	13.6	(18, 390)	0.149
Feeding system	0.06	12.7	(6, 128)	0.276

*SCC* somatic cell count; *df* degrees of freedom; *num* numerator; *den* denominator, *** (< 0.001); ** (0.01); *(0.05)

Multivariate pairwise comparison analysis, combined with effect size determination showed that each independent variable influenced the outcome at different levels. Milking frequency was highly associated with milk yield (*p* = 0.002). Mean milk yield from Ankole breed was statistically different (*p* = 0.002) from other breeds, whereas differences between other breeds were not significant (*p* > 0.05). While parity had shown positive influence on milk yield, multivariate pairwise comparisons did not show any difference between the two groups (*p* = 0.08). This significance might have happened by chance (Mordkoff 2019), since the Pillai’s Trace = 0.08 (Table 5). Results from Model 2 revealed that none of the independent variables was associated with SCC or antibiotic residues (*p* > 0.05).

**Table 5:** Determination of effect size of management factors associated with milk yield by multiple group comparisons analysis

Independent variable	Pillai value	F-value at df(6,128)	p-value	η^2^_*p*_	95% CI
Milking frequency				0.10	[0.01, 1.00]
Once—twice	0.15	3.8	0.002**		
Breeds				0.07	[0.00, 1.00]
Ankole—Crossbreed	0.14	3.5	0.017*		
Ankole—Friesian	0.15	3.9	0.007*		
Ankole—Jersey	0.14	3.7	0.012*		
Crossbreed—Friesian	0.09	2.3	0.202		
Crossbreed -Jersey	0.08	1.8	0.533		
Friesian—Jersey	0.07	1.8	0.575		
Parity				0.11	[0.01, 1.00]
Primiparous vs multiparous	0.08	1.908	0.084		
Body condition score				0.09	[0.01, 1.00]
Good—moderate	0.19	5.2	< 0.001***		
Good—poor	0.22	6.2	< 0.001***		
Moderate—poor	0.09	2.2	0.125		

*df* degrees of freedom; *η*^*2*^_*p*_ partial eta squared; *CI* confidence interval, *** (< 0.001); ** (0.01); *(0.05)

## Discussion

This study examined associations between prevalent management practices such as milking frequency, stage of lactation among the others on smallholder dairy farms in Rwanda, and milk quality and yield. Milk yield was found to be associated with factors such as cow breed, milking frequency, BCS and parity. Milk SCC were high, suggesting that some lactating cows had subclinical intramammary infections. Others had apparently been diagnosed with clinical mastitis and were undergoing antibiotic treatment, since 12.9% of milk samples tested positive for antibiotic residues.

Milk yield was positively associated with cow breed, where improved breeds produced more milk than the local breed, as found in previous studies in Ethiopia, Rwanda and Tanzania (Galukande et al. 2013; Gillah et al. 2014; Duguma 2020; Manzi et al. 2020). Breed was also associated with milk yield, but not with milk composition parameters, confirming previous findings (Sandoval-Castro et al. 2000; Wall and McFadden 2012). Poor animal management practices (such as insufficient and unbalanced diets, low disease control, poor housing, low water availability, and poor hygiene) are most likely the reason for the recorded low milk yield. It has been found that milking once a day dramatically reduces milk yield, by almost 22% of daily milk production (Stelwagen et al. 2013). Additionally, milk yield was associated with BCS and parity, which is in accordance with literature findings; cows with higher BCS produced more milk than those with low BCS, as expected (Roche et al. 2007) and multiparous cows produced more than primiparous cows, as found previously (Lee and Kim 2006). It has been suggested that the increase in milk yield in multiparous cows may be attributable to increasing cell development in the udder during subsequent pregnancies (Roche et al. 2007). This difference likely relates to the higher milk yield in multiparous cows (Azizi et al. 2009) and their distinct energy requirements for growth and lactation (Agnew and Yan 2000). Furthermore, cows with poor body conditions may not have sufficient energy reserves to extract nutrients from feed resources that support milk yield.

The average values of the milk composition parameters analysed were within the range reported in earlier studies conducted in Rwanda (Hirwa et al. 2017; Ayabagabo et al. 2021). However, Ayabagabo et al. (2021) observed greater fat content variations with season, associated with high fibre content in the feed, which increases acetate as well as volatile fatty acid content in milk. In the present study, milk lactose was not associated with feed/s used, feeding practices, breed, milking frequency, BCS or SCC, indicating that the farmers used similar farming practices. This is similar to findings in Kenya and Tanzania, but with higher values of milk composition parameters in different feeding systems, e.g. dairy cattle fed on Napier grass have a higher fat content in milk than those fed on natural pasture (Gillah et al. 2014; Kashongwe et al. 2017). Seasonal changes in the quantity and quality of feed and different agro-ecological zones can also affect milk yield and composition parameters (Hernández-Castellano et al. 2019; Ayabagabo et al. 2021). While this study did not examine the microbiological contamination of milk or the impact of psychrotrophic bacteria—biochemically active at low temperatures—on milk composition, previous research has demonstrated their effects on milk content through enzymatic activity (Hahne et al. 2019; Hu et al. 2018). The current findings showed that a few samples had very low fat and protein contents, at 1.1% and 2%, respectively, which may have been partly attributed to freezing temperatures and the influence of psychrotrophic bacterial activity on milk composition (Hu et al. 2018).

In contrast to previous findings (Stelwagen et al. 2013; Schwendel et al. 2015; Ramírez-Rivera et al. 2019), no relationship was found between milk yield and the feeds used, feeding system, or lactation stage. This could be partly explained by the fact that the majority of participating farmers (92.6%) use a cut-and-carry feeding system involving insufficient feeds of low quality, which results in low milk yield. This corroborates findings in a previous study by Maleko et al. (2018) that smallholder dairy farms in Tanzania that keep cows in a zero-grazing with cut and carry system have low milk productivity and poor milk composition. Similar to other tropical countries, land scarcity is the major contributing factor to feed shortage, as two-thirds of farmers are actually landless and cannot grow fodder in Rwanda (Kamanzi and Mapiye 2012). Additionally, most of our respondents had no previous experience of rearing dairy cattle, which might have contributed to low milk yield resulting from poor animal husbandry practices such as unbalanced diet, dirty shelters and disease. Lower milk yield means that farmers are unable to meet market demand (Nyamwaro et al. 2018), leading to lower consumption levels. In the present study, 34.2% of milk samples analysed had SCC above 300,000 cells/mL, i.e. were unacceptable for delivery to a milk collection centre in the Rwandan context (COMESA 2006). Intramammary infections are considered the most common reason for increased SCC in milk, but many other factors may contribute to high SCC. These include stage of lactation (in the beginning and in the end), parity, body condition, milking frequency and stress (Paape et al. 2007; Lianou et al. 2021). However, these factors were not associated with SCC in the present study. The high level of unacceptable SCC values obtained indirectly indicates a significant health risk to consumers. Consumption of milk with intramammary infections, indicated by high level of SCC, poses a public health risk, since potential pathogens such as *Staphylococcus* spp, *Escherichia coli*, *Campylobacter jejuni* etc., can cause several diseases other than food poisoning, e.g. chronic reactive arthritis (Mor-Mur and Yuste 2010), meningitis and abortion (D’Angelo et al. 2022).

The relatively high level of antibiotic residues in the milk samples analysed supports findings by other researchers (Alves et al. 2020) and could be due to ongoing or recent treatments for intramammary infections. Although we did not investigate whether the cows were undergoing certain treatments, recent studies have found that subclinical mastitis is one of the most common diseases affecting Rwandan dairy farms, with prevalence of more than 50% (Ndahetuye et al. 2020b; Mpatswenumugabo et al. 2017; Iraguha et al. 2015). In addition, the majority of farmers in our study did not keep records, which could be one reason why they may milk cows under treatment without respecting withdrawal times. It has been found that poor management practices and ineffective veterinary services lead to higher levels of antibiotic residues in milk (Rahman et al. 2021). A previous study in Rwanda revealed that 97.4% of farmers used antibiotics on-farm and nearly 60% of farmers bought antibiotics without a veterinary prescription (Manishimwe et al. 2017). However, we found no association with various management practices included in our statistical model. The proportion of samples with antibiotic residues in this study (12.9%) was around tenfold higher than in a previous study in Rwanda (1.3%) (Ndahetuye et al. 2020a). This difference might be due to the dilution effect, since in the current study milk samples were collected from individual cows, while Ndahetuye et al. (2020a) collected samples of bulk tank milk. When milk samples are pooled, the concentrations of antibiotic residues could be diluted to undetectable levels (Rahman et al. 2021). When individual milk samples are collected from each cow, milk from individual cows treated with antibiotics before sample collection may contain high levels of antibiotic residues (Rahman et al. 2021).

Poor detection facilities and lack of a proper monitoring system for antibiotic residues in foods prevent full assessment (Pokharel et al. 2020). The microbial-based detection method used in the present study is cost-effective and able to cover the entire antibiotic spectrum with a single test (Pikkemaat 2009). The detection rate (12.9%) was much lower than in a study in Ghana by Aning et al. (2007), who found antibiotic residues in 35.5% of samples from peri-urban areas, and in a study in Tanzania by Kurwijila et al. (2006), where 36% of milk samples tested positive for antibiotic residues. The presence of antibiotic residues in raw milk affects the quality of dairy products by reducing the growth of starter culture, milk curdling, and cheese ripening, as well as flavour production (Virto et al. 2022). There are also health risks associated with consumption of milk containing antibiotic residues, including the development of antimicrobial resistance, hypersensitivity reactions, toxicities and cancer (Rahman et al. 2021).

In conclusion, this study provided new information on management practices on smallholder dairy farms in Rwanda and their relationship with milk yield and quality. Since our findings corroborate with previous studies in developing countries, the current results can act as baseline information for policymakers and researchers, e.g. in risk analysis on public health threats associated with antibiotic residues in food. Milk yield was found to be associated with some management practices, so smallholder farmers in Rwanda need to be educated on best farming practices, including suitable feed rations and milking frequencies. Milk composition parameters were not associated with management practices, but routine analysis of milk composition parameters should be established to detect any milk adulteration. High levels of SCC and presence of antibiotic residues in milk suggest constant exposure of consumers, with potential health risks. Extension services should support farmers’ cooperatives or smallholder farmers to improve feeding practices and exploit the production capacity of improved breeds. Routine testing for SCC and antibiotic residues at milk collection centres is important to ensure high quality and safety of raw milk and safeguard consumers. Finally, proper farm management would increase both the quantity and quality of cow milk, thus helping to meet increasing market demand and securing human livelihoods.

## Study limitations

The study highlights several limitations that support the context of the findings and inform future research directions (Ross and Zaidi 2019). First, the cross-sectional design cannot establish direct causality between independent variables and milk yield, but existing literature supports these associations. Second, due to the study’s involvement in a larger project on child undernutrition in Rwanda, milk samples were frozen for later molecular analysis in Sweden. Despite some values for fat and protein content falling below normal, mean values remained within range, suggesting minimal impact from freezing. Lastly, the sample size was limited to 156 households with lactating cows out of 601, to prevent bias in assessing child stunting. The current findings are relevant due to methodological reproducibility and consistency with previous studies. Therefore, future studies should use longitudinal designs and analyze milk content soon after milking, without freezing, to improve accuracy and causal understanding.

## Data Availability

Not applicable. Data were not deposited in an official repository. No new datasets were created.
